# DNA Replication Determines Timing of Mitosis by Restricting CDK1 and PLK1 Activation

**DOI:** 10.1016/j.molcel.2018.05.026

**Published:** 2018-07-05

**Authors:** Bennie Lemmens, Nadia Hegarat, Karen Akopyan, Joan Sala-Gaston, Jiri Bartek, Helfrid Hochegger, Arne Lindqvist

**Affiliations:** 1 Department of Cell and Molecular Biology, Karolinska Institutet, Stockholm, Sweden; 2 Division of Genome Biology, Department of Medical Biochemistry and Biophysics, Karolinska Institutet and Science for Life Laboratory, Stockholm, Sweden; 3 Genome Damage and Stability Centre, University of Sussex, Brighton, UK; 4 Danish Cancer Society Research Center, Copenhagen, Denmark

**Keywords:** cell cycle, S phase, G2 phase, mitosis, replication checkpoint, DNA replication, CDK1, PLK1

## Abstract

To maintain genome stability, cells need to replicate their DNA before dividing. Upon completion of bulk DNA synthesis, the mitotic kinases CDK1 and PLK1 become active and drive entry into mitosis. Here, we have tested the hypothesis that DNA replication determines the timing of mitotic kinase activation. Using an optimized double-degron system, together with kinase inhibitors to enforce tight inhibition of key proteins, we find that human cells unable to initiate DNA replication prematurely enter mitosis. Preventing DNA replication licensing and/or firing causes prompt activation of CDK1 and PLK1 in S phase. In the presence of DNA replication, inhibition of CHK1 and p38 leads to premature activation of mitotic kinases, which induces severe replication stress. Our results demonstrate that, rather than merely a cell cycle output, DNA replication is an integral signaling component that restricts activation of mitotic kinases. DNA replication thus functions as a brake that determines cell cycle duration.

## Highlights

• Double-degron system allows rapid protein degradation and DNA replication control
• DNA replication restrains PLK1 and CDK1 activities until S/G2 transition
• DNA replication, via CHK1/p38 signaling, prevents CDK1-driven DNA damage
• DNA replication functions as a brake that determines the timing of mitosis

## Graphical Abstract

**Figure:**
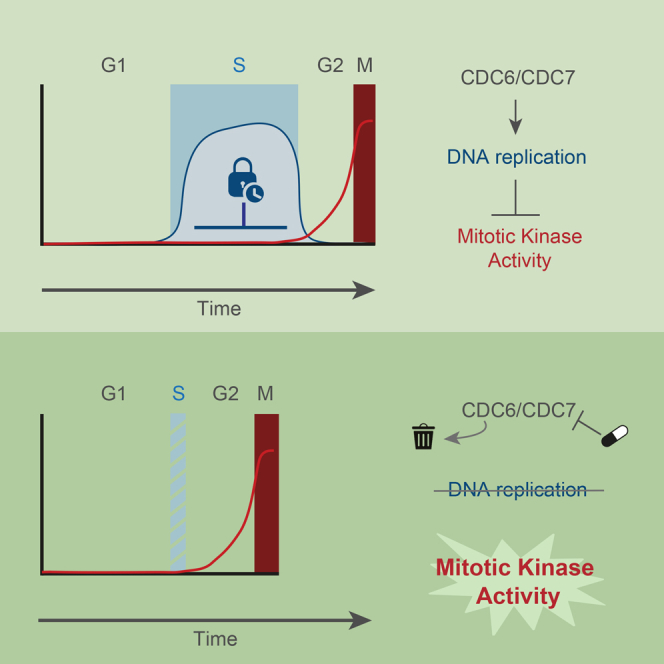

## Teaser

Proliferating cells need to duplicate their genome before they divide. Lemmens et al. find that DNA replication determines the timing of cell division by restricting activation of mitotic kinases. This mechanism ensures that DNA replication and cell division are separated in time and prevents replication stress.

## Introduction

Cell proliferation is critical to the propagation of life and in eukaryotes is based on mitotic cell division. Mitotic cell division requires two basic steps: first, cells need to replicate their DNA, and second, cells need to divide the replicated genome to daughter cells. Strict separation of these steps in time is critical as division of partially duplicated chromosomes causes genome instability and cell death (Boddy et al., 1998, Piatti et al., 1995, Tercero et al., 2000). Furthermore, activation of mitotic factors during DNA replication causes replication fork instability and DNA damage (Beck et al., 2010, Neelsen et al., 2013). How cells coordinate genome duplication and mitosis thus constitutes a fundamental question in cell cycle control, which has major consequences for both proliferation and genome stability.

Almost 30 years ago, Hartwell and Weinert introduced the concept of a checkpoint and described it as a safety mechanism that prevents cell division if DNA replication is not complete (Hartwell and Weinert, 1989). However, empirical data supporting an intrinsic DNA replication checkpoint proved hard to obtain, as treatments to block DNA replication fork progression are prone to cause DNA damage, evoking secondary responses that halt mitotic progression (Kurose et al., 2006, Natsume et al., 2017). The concept of a checkpoint soon expanded and became increasingly used to depict a cell cycle block in response to DNA damage and other cellular stresses. Stress-induced checkpoints have been widely studied, which has led to the identification of well-conserved signaling kinases, such as CHK1 and p38 (Bulavin et al., 2002, Reinhardt and Yaffe, 2009). Meanwhile, the discovery of autonomous biochemical oscillators in yeast cells and de-nucleated *Xenopus* egg extracts provided alternative models that could explain orderly cell cycle progression without the need of a DNA replication checkpoint (Haase and Reed, 1999, Newport and Kirschner, 1984, Stern and Nurse, 1996). That is, self-regulating oscillators could in principle ensure sufficient time to finish DNA replication before mitosis is triggered.

**Figure 1:**
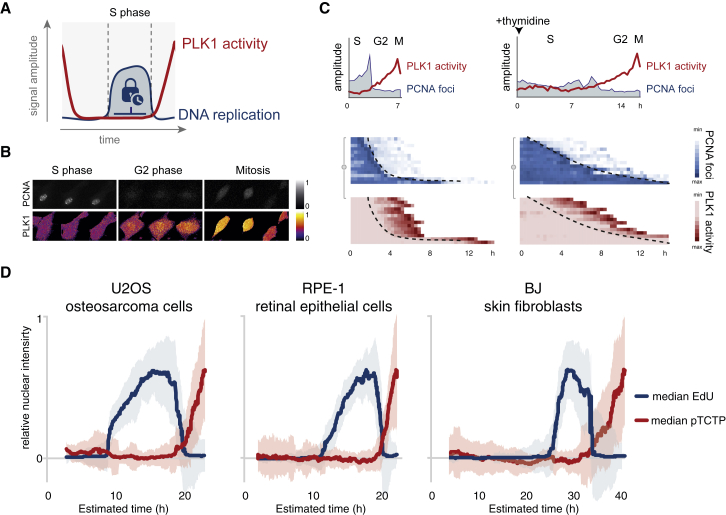
Plk1 Activation Correlates to Completion of DNA Replication (A) Schematic of hypothesis. (B) Example of RPE cell expressing PLK1-FRET and PCNA-cb in S phase, G2 phase, and mitosis. Time between images is 20 min. Please note negative correlation between nuclear PLK1 activity and presence of PCNA-cb foci. (C) S phase cells expressing PCNA-cb foci were imaged every 20 min and either mock treated or exposed to 2.5 mM thymidine. (Top) Single-cell examples of PLK1 activity and PCNA foci quantifications are shown. (Bottom) Color-coded heatmap of PLK1 activity and PCNA-cb quantifications of multiple single cells are shown. Dotted line highlights temporal correlation between DNA replication completion and PLK1 activation. Further characterization of thymidine-induced S phase arrest is described in Figure S1. (D) U2OS, RPE, or BJ cells were fixed after a 1-hr EdU pulse and monitored by high-content microscopy. Cells were sorted based on cyclin A2 levels and nuclear size and plotted versus estimated time (Akopyan et al., 2016). Graphs show moving median and SD of EdU signal and pTCTP signal from >1,600 single cells. EdU incorporation is used to measure DNA replication in single cells. pTCTP signal is corrected by treating a control population with the Plk1 inhibitor BI2536. A stepwise scheme of simultaneous cell cycle and TCTP phosphorylation analysis is described in Figure S2.

The key to understand how DNA replication and cell division are separated is to understand how cell division is initiated. Mitotic entry is driven by the kinases CDK1 and PLK1, which through multiple feedback loops enhance each other’s activity (Lindqvist et al., 2009). Activation of CDK1 depends on CDK2-mediated accumulation of mitotic inducers, such as PLK1, which at a threshold level can induce a switch-like activation and mitotic entry. The threshold is defined by factors opposing CDK1 activation, including PP1 and PP2A phosphatases and Wee1/Myt1 kinases (Hégarat et al., 2016). In addition, checkpoint kinases as CHK1 and p38-MK2 can raise the activation threshold through phosphorylation of multiple CDK regulators (Reinhardt and Yaffe, 2009), effectively suppressing CDK activity in the presence as well as absence of exogenous DNA damage (Beck et al., 2010, Rodríguez-Bravo et al., 2007, Sørensen et al., 2004, Warmerdam et al., 2013). However, if and how unperturbed DNA replication is integrated into this regulatory circuit remains unknown. We previously showed that both CDK1 and PLK1 are activated when the bulk of DNA replication is completed at the S/G2 transition (Akopyan et al., 2014). These observations resurged the prospect of the original checkpoint model and prompted us to test whether DNA replication restricts mitotic kinase activation (Figure 1A).

Here, we generated a double-degron system to rapidly deplete the essential DNA replication-initiation factor CDC6 and show that untransformed human cells shorten the cell cycle and prematurely enter mitosis in the absence of DNA replication. Using RNAi and inhibitors to independently target DNA replication licensing or firing, we find similar results in cancer cells. We also find that abrogating CHK1 activity in transformed cells, or CHK1 and p38 activity in untransformed cells, enhances CDK activation specifically upon G1/S transition, supporting the notion of a DNA replication checkpoint in human cells. While checkpoint inhibition causes premature activation of CDK2, PLK1, and CDK1, we find the latter most harmful and causing severe DNA replication stress.

Our results indicate that DNA replication is not only an output but also an integral signaling component of the cell cycle oscillator and is a major determinant for the timing of mitosis. DNA replication, through checkpoint kinase signaling, coordinates the cell cycle by allowing a slow buildup of CDK2 activity while suppressing CDK1 and PLK1 activation until S phase completion.

## Results

### Nuclear PLK1 Activation Marks Completion of DNA Replication at S/G2 Transition

In order to simultaneously monitor DNA replication and mitotic kinase activation, we generated untransformed but immortalized retinal pigment epithelial (RPE) cell lines expressing a marker for DNA replication (proliferating cell nuclear antigen [PCNA]-cb) and a sensor for PLK1 target phosphorylation (PLK1-FRET). In agreement with our previous findings in U2OS cancer cells, PLK1 target phosphorylation remained undetectable during active DNA replication and only appeared when the bulk of PCNA foci disappeared (Figure 1B). The close correlation between PCNA foci disappearance and Plk1-mediated phosphorylation was retained when completion of DNA replication was delayed by addition of thymidine (Figures 1C, S1A, and S1B). To test PLK1-dependent phosphorylation of an endogenous target, we next assessed TCTP-pS46 staining (Cucchi et al., 2010) by quantitative immunofluorescence. To estimate temporal kinetics from fixed asynchronous cell populations, we sorted single cells based on cyclin A2 content, DNA content, and nuclear size (Akopyan et al., 2014, Akopyan et al., 2016). DNA replication was assessed by measuring EdU nucleotide incorporation immediately before fixation, and PLK1 activity was determined by PLK1-dependent TCTP-pS46 staining using a low dose of the PLK1 inhibitor BI2536 (Figure S2A). We note that there is a close correlation between the disappearance of EdU staining and the appearance of BI2536-sensitive TCTP-pS46 staining in U2OS, RPE, and early-passage primary fibroblasts (Figures 1D, S2A, and S2B). The robust coupling between completion of S phase and activation of PLK1 as assessed by both fixed and live readouts suggests that PLK1 activation can function as a marker for the S/G2 transition in human cells.

### A Double-Degron System to Prevent DNA Replication Licensing

**Figure 2:**
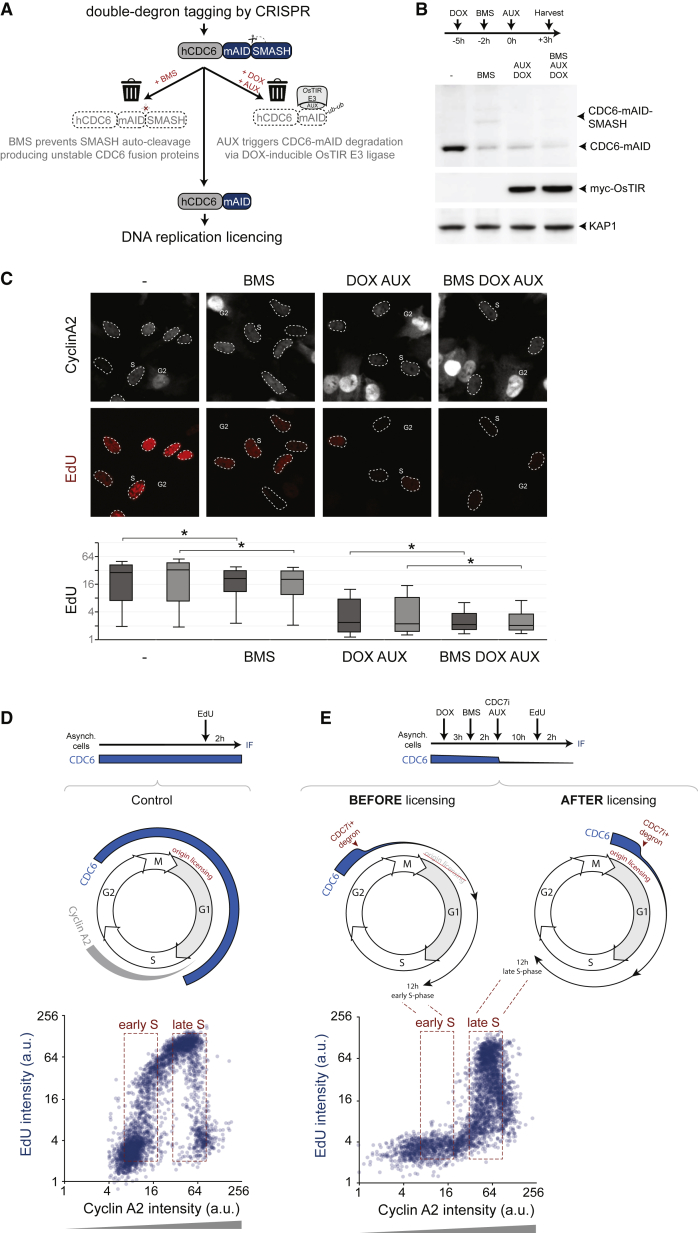
Suppression of DNA Replication Initiation by Targeting CDC6 and CDC7 (A) Schematic of approach to target CDC6^d^ degradation by a double-degron system. (B) RPE CDC6^d^ cells were treated as outlined, and cell lysates were probed against indicated proteins. (C) RPE CDC6^d^ cells were treated as in (B) yet fixed 48 hr post-AUX addition. EdU was added 1 hr prior to fixation. Upper panel shows representative images of EdU and cyclin A2 expression, and examples of G2 or S phase cells (dotted circles) are indicated. EdU incorporation is used to measure DNA replication in single cells. Lower panel depicts quantification of EdU intensities of S phase cells, as determined by nuclear cyclin A2 levels. Boxplots indicate 10, 25, 50, 75, and 90^th^ percentiles of at least 200 cells per condition. ^∗^ indicates p < 0.01; Student’s t test. Light or dark gray bars represent data from 2 independent CDC6^d^ clones. (D) RPE CDC6^d^ cells were treated as outlined in top panel, and anticipated cell cycle events are shown in middle panel. EdU incorporation is used to measure DNA replication in single cells. Lower graph depicts quantification of EdU staining versus cyclin A2 levels to detect early and late S phase cells. Please note rapid induction of EdU incorporation in early S phase cells. (E) RPE CDC6^d^ cells were treated as outlined in top panel, and anticipated cell-cycle-dependent effects of combined CDC6^d^ degradation and CDC7 inhibition are shown in middle panels. Lower graph depicts quantification of EdU staining versus cyclin A2 levels to detect early and late S phase cells. Please note that targeting CDC6 and CDC7 specifically inhibits initiation of DNA replication in early S phase cells.

To assess whether PLK1 activation is restricted by DNA replication, we sought to analyze cells that progressed through the cell cycle without replicating DNA. In yeast, mutants in the DNA replication licensing factor Cdc6 can efficiently suppress DNA replication without blocking cell cycle progression (Muzi Falconi et al., 1996, Piatti et al., 1995). We therefore set out to generate a genetic system that would allow rapid degradation of CDC6 in untransformed human cells. We used p53^−/−^ RPE cells to avoid a licensing checkpoint (Nevis et al., 2009, Shreeram et al., 2002) and labeled both endogenous CDC6 alleles with an optimized double-degron tag. The double degron is based on two protein degradation tags: mAID (Nishimura et al., 2009) and SMASh (Chung et al., 2015), allowing rapid depletion of endogenously tagged CDC6^d^ (Figures 2A, 2B, S3A, and S3B; STAR Methods). CDC6^d^ degradation using both mAID and SMASh prevented EdU incorporation more efficiently than using mAID or SMASh alone, showing an additive effect of both degradation tags on suppressing DNA replication (Figures 2C and S3B). After 48 hr CDC6^d^ degradation, cyclin A2 expression and CDK-mediated phosphorylation of lamin A/C pS22 was not reduced, indicating that cells remained engaged in the cell cycle (Figures S3C and S3D). Whereas mitotic cells were abundant, they showed half the DNA content of their control counterparts and few anaphase figures, as expected for cells entering mitosis without sister chromatids (Figures S3E–S3G). Taken together, our results show that the CDC6^d^ double-degron system can be used to suppress DNA replication.

### Suppression of DNA Replication Initiation in Unsynchronized Human Cells

We next pursued to study cell cycle progression in the absence of DNA replication. We aimed to prevent initiation of replication, as perturbation of ongoing replication leads to DNA damage and cell cycle arrest (Natsume et al., 2017). Meanwhile, to avoid secondary effects due to continued proliferation with incomplete DNA replication, we sought to detect the immediate effects of suppressed initiation of DNA replication. To this end, we combined CDC6^d^ degradation and a CDC7 inhibitor, targeting both origin licensing and origin firing, the two main events necessary for initiation of DNA replication. DNA replication licensing by CDC6 can in principle start as soon as CDK activities are reset at anaphase. Cells depleted of CDC6^d^ before mitotic exit should thus be impaired in DNA replication initiation. In contrast, G1 or S phase cells already harbor licensed origins and thus should remain DNA replication competent despite CDC6^d^ degradation. To test this prediction, we monitored EdU incorporation between 10 and 12 hr after targeting CDC6, CDC7, or both CDC6 and CDC7. We reasoned that, at this time, G2 cells would have progressed through mitosis and recently entered S phase, whereas G1 cells would have progressed to late S phase (Figures 2D and 2E). To independently monitor DNA replication and cell cycle position, we plotted EdU incorporation versus cyclin A2 content of single cells. The gradual accumulation of cyclin A2 levels from the G1/S border allows identification of cell cycle position within S phase (Akopyan et al., 2014). As predicted, EdU incorporation was effectively abrogated in early S phase cells, whereas it was sustained in late S phase cells (Figures 2D and 2E). This shows that our setup efficiently prevents initiation of DNA replication, while allowing ongoing DNA replication to continue.

### DNA Replication Determines Cell Cycle Duration

**Figure 3:**
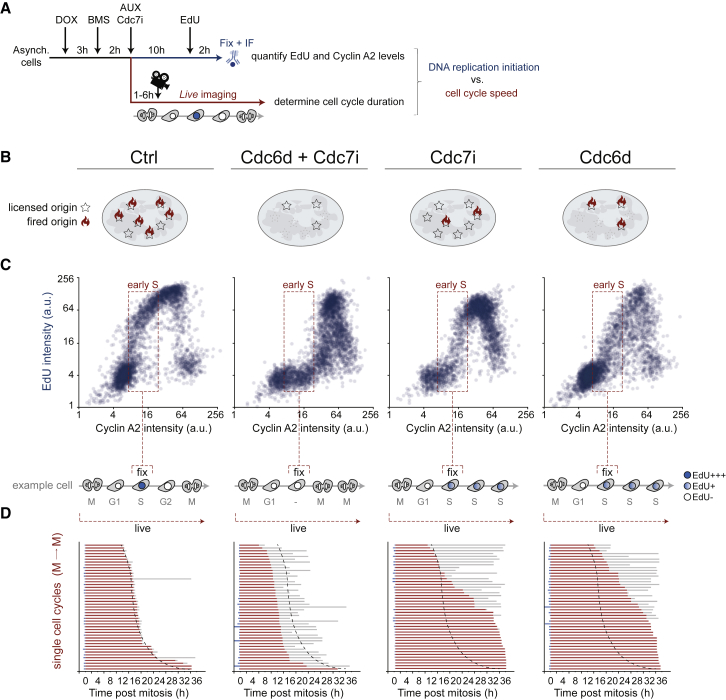
DNA Replication Controls Cell Cycle Duration (A) Asynchronous RPE CDC6^d^ cells were treated as outlined and either fixed after a 2-hr pulse of EdU or followed by time-lapse microscopy. The level of DNA replication initiation, measured by EdU incorporation in single early S phase cells, is correlated directly to cell cycle speed. (B) Schematic of anticipated effect of CDC6^d^ degradation and/or CDC7 inhibition. (C) Cells were treated as outlined in (A) and (B), and nuclear EdU intensity was plotted versus nuclear cyclin A2 levels to monitor DNA replication during cell cycle progression. Please note that combined CDC6 degradation and CDC7 inhibition prevents EdU incorporation in early S phase cells. Lower panels illustrate a single cell entering mitosis after CDC6 degradation and/or CDC7 inhibition (i.e., upon addition of AUX/CDC7i). Illustration depicts relative timings of empirical tests (fix or live) as well as anticipated cell fate. (D) Tracks of single cells passing through mitosis within 6 hr after addition of auxin or CDC7i were followed from mitosis to the following mitosis. Grey bars indicate mitosis, and red bars indicate interphase. See Figure S4 for example images of mitotic cells.

To study the direct consequence of failed DNA replication initiation, we used time-lapse microscopy to follow single cells that entered mitosis shortly after destruction of CDC6^d^ and inhibition of CDC7 (Figures 3A and S4; Video S1). In parallel, we treated cells with EdU and stained for cyclin A to detect efficiency of DNA replication in S phase (Figures 3A–3C). Combined CDC6^d^ depletion and CDC7 inhibition decreased median cell cycle duration by ∼4 hr (−25%) and allowed cells to re-enter mitosis as early as 6–8 hr after cell division (Figure 3D). Initiation of DNA replication thus constitutes a major brake on cell cycle progression.

Video S1. Example of RPE CDC6^d^ Cell Entering Mitosis Shortly after Treatment with DOX/BMS/AUX/Cdc7i and Re-entering Mitosis Less Than 10 hr Later, Related to Figure 3 One second in the movie corresponds to 100 min.

We find that only a limited amount of DNA replication is sufficient to delay the cell cycle. After CDC6^d^ depletion or CDC7 inhibition alone, EdU incorporation is suppressed but still detectable in early S phase cells, while double treatment effectively abolished new synthesis of DNA (Figures 3B and 3C). The need for combined CDC6^d^ depletion and CDC7 inhibition to efficiently suppress DNA replication could in part be due to compensatory mechanisms that enhance firing in genomic regions where origins are sparse (Fragkos et al., 2015, Ozeri-Galai et al., 2011) or due to the fact that CDK activity can complement CDC7 to fire a restricted set of origins (Rainey et al., 2017). Importantly, CDC6^d^ depletion or CDC7 inhibition alone postponed mitotic entry, increasing median cell cycle duration by ∼10 hr (+70%; Figure 3D). The limited availability of licensed or fired origins is expected to delay DNA replication completion, which in turn could postpone mitotic entry. Our results are in agreement with a threshold model in which low levels of DNA replication are sufficient to prolong the cell cycle while a complete block of DNA replication leads to premature mitosis.

### DNA Replication Controls Activation of Plk1

**Figure 4:**
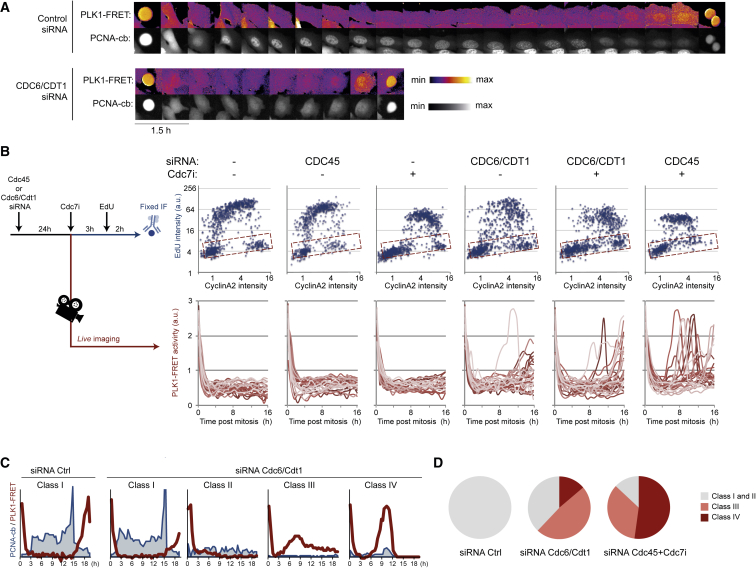
DNA Replication Restricts Activation of PLK1 (A) Examples of U2OS cells expressing PLK1-FRET and PCNA-cb followed in the absence or presence of combined CDC6/CDT1 RNAi. Time between images is 45 min. Please note absence of PCNA foci despite proficient PCNA-cb expression and nuclear PLK1 activation within 5 hr. (B) U2OS cells expressing PLK1-FRET were treated as outlined in left panel and fixed after a 2-hr pulse of EdU or followed by time-lapse FRET microscopy. (Top) EdU quantification of >600 single cells; nuclear EdU intensity was plotted versus nuclear cyclin A2 levels to monitor DNA replication during cell cycle progression. Outlined boxes indicate EdU-negative cells; please note that the three most right conditions generate cells that have intermediate cyclin A2 levels but fail to incorporate EdU, which corresponds to the conditions that have premature PLK1 activation. (Bottom) Live-cell traces of single cells that entered mitosis within 2 hr upon addition of CDC7i were followed for 16 hr. Graphs show quantified PLK1 activity of 25 single cells. (C) Representative single-cell traces of the different classes observed upon Cdc6/Cdt1 co-depletion. U2OS cells expressing PLK1-FRET and PCNA-cb were transfected with control or Cdc6/Cdt1 small interfering RNA (siRNA) for 24 hr and mitotic cells were followed by live-cell imaging. The distinct classes include (class I) cells similar to controls, having discrete PCNA foci and PLK1-FRET activity only upon PCNA foci resolution, (class II) cells maintaining dim PCNA foci and no PLK1-FRET activity, and (classes III and IV) cells with no or very few PCNA foci and premature PLK1-FRET activity (4–16 hr post), which either undergo S/G2 arrest (class III) or premature mitosis (class IV). (D) Pie charts depict distribution of classes I–IV among cells depicted in (B). Whereas targeting licensing or firing can cause premature mitosis, we find the latter approach to be most effective.

To study the impact of DNA replication on activation of PLK1, we targeted origin licensing (by depleting licensing factors CDC6 and CDT1) or origin firing (by combined CDC45 depletion and CDC7 inhibition) in U2OS PLK1-FRET PCNA-cb cells. We find that, after interfering with either DNA replication licensing or origin firing, a subset of cells showed PLK1 activation 4–6 hr after mitosis (Figures 4A, 4B, and S5A). Similarly, some cells entered mitosis 6–8 hr after cell division, in contrast to control cells, in which the shortest time between two cell divisions was 15 hr. Premature PLK1 activation correlated to the absence of PCNA-cb foci and abolished EdU incorporation in S phase cells, indicating that premature PLK1 activation depends on suppression of DNA replication (Figures 4A–4D and S5B). In agreement with a threshold model, intermediate reduction of EdU incorporation or PCNA foci delayed PLK1 activation compared to control cells (Figure 4C). Our data suggest that DNA replication restricts activation of the mitotic kinase PLK1 and that completion of DNA replication functions as a trigger for PLK1 activation.

### CHK1/p38 Signaling Represses PLK1 Activity and Toxic CDK1 during DNA Replication

**Figure 5:**
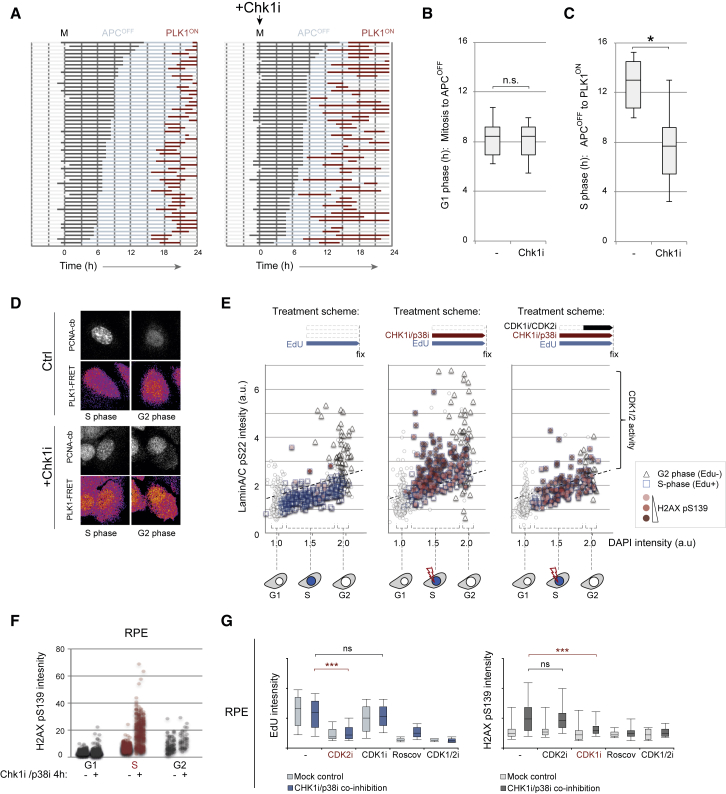
DNA Replication Restricts PLK1 and CDK1/2 Activity to Prevent Replication Stress (A) Asynchronous U2OS cells expressing PLK1-FRET and APC/C^Cdh1^ substrate reporter were mock treated or treated with 50 nM CHIR-124 at mitosis (±1 hr) and followed by time-lapse microscopy for 24 hr. Each line represents a single cell showing expression of APC/C^Cdh1^ substrate probe (light blue) and/or PLK1 activity (red). Visual appearance of nuclear GEM-RED signal (i.e., stabilization of the APC/C^Cdh1^ substrate probe) was used to assess APC/C inactivation at the G1/S transition (APC^OFF^). Detection of nuclear PLK1 FRET signal was used to assess PLK1 activation at the S/G2 transition (PLK1^ON^). (B) Boxplot depicts G1 phase duration of 50 single cells, as determined by the time observed between mitosis and APC/C^Cdh1^ substrate appearance at the G1/S transition. Boxplots indicate 10, 25, 50, 75, and 90^th^ percentiles. *n.s.* indicates p > 0.5; Student’s t test. (C) As in (B), yet boxplot depicts S phase duration as determined by the time observed between APC/C^Cdh1^ substrate appearance and the detection of nuclear PLK1 activity. Boxplots indicate 10, 25, 50, 75, and 90^th^ percentiles. ^∗^ indicates p < 0.01; Student’s t test. (D) Images illustrate representative cells in S phase (13 hr post-mitosis) or G2 phase (17 hr post-mitosis). (E) Asynchronous RPE cells were treated with EdU, CHIR-124 (Chk1i), and SB202190 (p38i) for 2 hr and during the last hour with RO-3306 (CDK1i) and NU6140 (CDK2i) as indicated. EdU is used to mark S phase cells and directly correlate the presence of DNA replication with CHK1i-induced CDK activation and DNA damage. Graphs show DAPI, lamin A/C pS22, and H2AX pS139 for >800 cells measured by high-content microscopy. Single-cell illustrations below the graph indicate cell cycle phases and presence of DNA replication stress. (F) RPE cells were treated with CHIR-124 (CHK1i) and SB202190 (p38i) as indicated. To define S phase, cells were incubated with EdU 1 hr prior to fixation. Panels show quantifications of high-content microscopy for H2AX pS139 in single cells. Cell populations were separated according to DAPI and EdU intensity. (G) RPE cells were treated with CHIR-124 (CHK1i), SB202190 (p38i), NU6140 (CDK2i), RO3306 (CDK1i), CDK1/2 inhibitor III (CDK1/2i), and Roscovitine as indicated for 4 hr. Before fixation, cells were incubated for 1 hr with EdU. Boxplots show 90, 75, 50, 25, and 10^th^ percentiles of EdU signal or H2AX pS139 signal of EdU positive cells, as assessed by high-content microscopy. ^∗∗∗^ indicates p < 0.001, and ns indicates p > 0.1; Student’s t test.

The function of replication intermediates as direct inhibitors of mitosis via constitutive checkpoint activation is a long-standing yet unproven hypothesis in the cell cycle field (Enoch and Nurse, 1991, Hartwell and Weinert, 1989). Our finding that DNA replication initiation postpones cell cycle progression predicts the need for factors that can sense ongoing DNA replication and suppress the activity of mitotic kinases. Good candidates for such signaling factors are DNA damage checkpoint kinases, as they are known to control DNA replication fidelity during unperturbed growth and inhibit PLK1 activity upon ectopic DNA damage (Smits et al., 2000, Sørensen and Syljuåsen, 2012). Indeed, we find that two structurally unrelated CHK1 inhibitors cause premature PLK1 activation in replicating U2OS cells (Figure S6A). Notably, CHK1 inhibition did not affect progression through G1 phase, but uncoupled nuclear PLK1 target phosphorylation from on-going DNA replication, causing interphase cells to reach high PLK1-FRET levels in the presence of PCNA-cb foci (Figures 5A–5D). Similarly, a live-cell CDK1/2-activity sensor rapidly became saturated in U2OS cells containing PCNA-cb foci (Figures S6B and S6C). CHK1 can function synergistically with the p38-MK2 axis (Dietlein et al., 2015), and combined CHK1/p38 inhibition increased CDK1/2-mediated phosphorylation of lamin A/C in RPE cells (Figure 5E). These data support the notion that mitotic kinases are actively suppressed during S phase.

Loss of CDK1/2 regulation is highly genotoxic and causes DNA replication stress (Sørensen and Syljuåsen, 2012). Accordingly, inhibition of CHK1/p38 leads to phosphorylation of the DNA damage marker H2AX specifically in replicating cells (Figures 5E, 5F, and S6D). A short pulse of CDK activity is likely sufficient to induce DNA damage, as contrary to CDK inhibition 1 hr after CHK1/p38 inhibition, simultaneous addition of all inhibitors abolished H2AX phosphorylation (Figures 5E, 5G, S6E, and S6F). Whereas addition of a CDK2 inhibitor decreased EdU incorporation in S phase cells, inhibition of CDK1 suppressed phosphorylation of H2AX (Figures 5G and S6F). Although this does not rule out overlapping functions of CDK1 and CDK2, it suggests that CDK1 activity is the major toxic component for replicating cells. Thus, CDK2 activity stimulates DNA replication (Coverley et al., 2002, Walter, 2000), which in turn activates checkpoint kinases to restrict CDK1 and PLK1 activity. As a consequence, DNA replication is part of a feedforward system that restricts mitotic kinase activation to prevent replication stress.

### DNA Replication Checkpoint Acts after G1/S Transition to Limit CDK Activation

**Figure 6:**
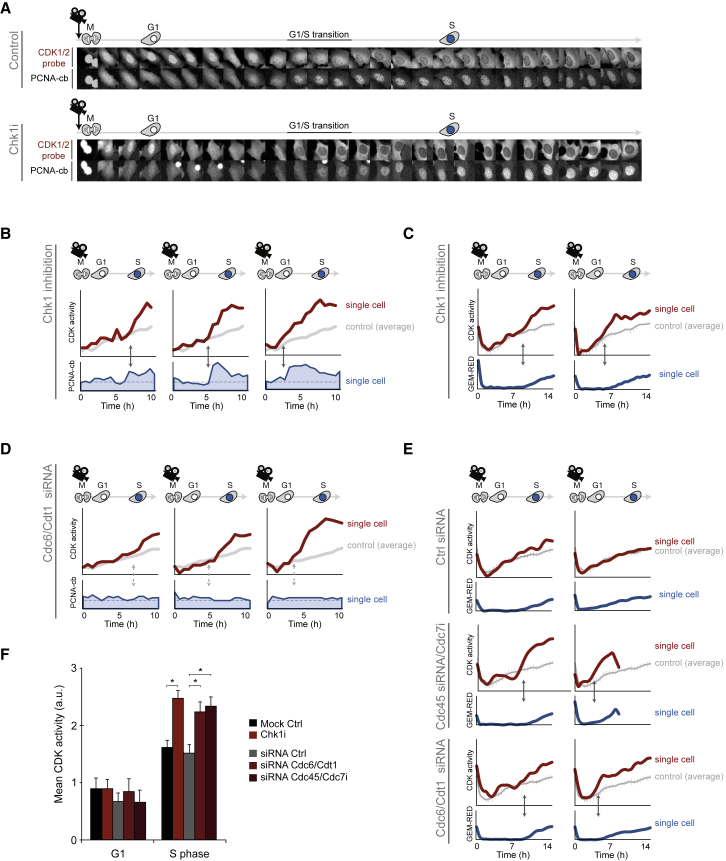
DNA Replication Limits CDK1/2 Activation upon S Phase Entry (A) Single U2OS cells were filmed upon mitotic exit, and cells that were ±1 hr from mitosis upon addition of CHIR-124 (CHK1i) were selected for analysis. Montages depict an example cell expressing CDK1/2 activity sensor and PCNA-cb followed by time-lapse microscopy. Time between images is 45 min. Note the rapid cytoplasmic translocation of the CDK1/2 sensor at the G1/S transition in the presence of CHK1i. Please see Figure S6C for readout details. (B) Quantification of single cells imaged as in (A). Red line indicates relative CDK1/2 activity, and blue line indicates PCNA-cb intensity variation as a surrogate measurement for DNA replication. Grey lines show average CDK1/2 activity of control cells. To illustrate concurrence at G1/S, three single cells with different G1 lengths are depicted. (C) U2OS cells expressing CDK1/2 activity sensor and APC/C^Cdh1^ substrate (GEM-RED) after CHIR-124 treatment and followed as in (A). To illustrate concurrence at G1/S, two single cells with different G1 lengths are depicted. (D) Quantification of single cells imaged as in (A) yet 24 hr after transfection of Cdc6 and Cdt1 siRNA. Red line indicates relative CDK1/2 activity, and blue line indicates PCNA-cb intensity variation as a surrogate measurement for DNA replication. Grey lines show average CDK1/2 activity of control cells. (E) U2OS cells expressing CDK1/2 activity sensor and APC/C^Cdh1^ substrate (GEM-RED) treated and followed as in (D). To illustrate concurrence at G1/S, two single cells with different G1 lengths are depicted per condition. (F) Quantification of CDK1/2 reporter of cells monitored as in (C) and (E). G1 is defined as 1 hr before and S phase as 3 hr after appearance of APC/C^Cdh1^ substrate reporter. Graph shows mean and SD of 14 cells per condition. ^∗^p < 0.04; Student’s t test.

**Figure 7:**
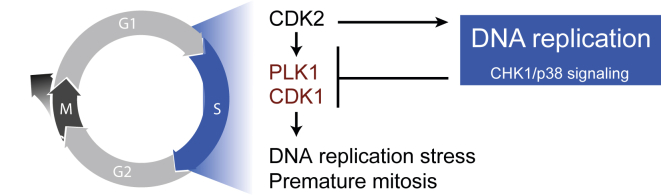
Model for Integral Role of DNA Replication in Human Cell Cycle Wiring Proposed model in which DNA replication constitutes a functional output as well as an integral component of an incoherent feedforward loop in human cell cycle signaling. DNA replication generates a CHK1- and p38-dependent signal that prevents premature mitosis and CDK1-driven DNA damage in S phase.

A prediction of such a system is that DNA replication continuously restricts CDK activity. We therefore sought to monitor CDK activity as cells entered S phase. A *live* sensor for CDK1/2 activity shows a gradual increase, starting from before S phase is initiated (Cappell et al., 2016; Figure 6A). In the presence of CHK1 inhibition, we detect no difference in CDK1/2 sensor localization through G1 phase (Figures 6A–6C). However, we detect enhanced CDK activity from the moment cells enter S phase as estimated by appearance of PCNA-cb foci (Figures 6A and 6B) or by expression of an anaphase-promoting complex (APC)/C^Cdh1^ substrate reporter (Cappell et al., 2016; Figure 6C). Similarly, blocking DNA replication licensing or DNA replication firing causes elevated CDK activity upon S phase entry (Figures 6D–6F). We also detect elevated CDK1/2-mediated phosphorylation of Lamin A/C pS22 in cells in which DNA replication initiation is prevented by targeted destruction of CDC6^d^ (Figures S7A–S7C). Thus, rather than merely a consequence of cell cycle signaling (Krude et al., 1997), DNA replication coordinates gradual CDK activation throughout S phase with activation of mitotic kinases PLK1 and CDK1 after completion of S phase (Figure 7).

## Discussion

Separation of DNA replication and cell division lies at the heart of the cell cycle. Several not mutually exclusive models for how cells ensure completion of DNA replication before mitosis have been proposed (reviewed in Uhlmann et al., 2011). These models include (1) a slow buildup of CDK activity coupled to different thresholds for starting DNA replication and mitosis (Stern and Nurse, 1996), (2) different cyclin proteins appear at given times and cellular locations (Murray, 2004), (3) transcriptional oscillator networks (Haase and Reed, 1999; but see also Rahi et al., 2016), and (4) a checkpoint that restricts mitosis before DNA replication is completed (Hartwell and Weinert, 1989). Our results suggest that, in human cells, DNA replication is a major determinant of cell cycle duration and timing of mitosis. We propose that DNA replication functions as a continuous brake with the possibility to separate and coordinate different cell cycle activities.

We find that severe suppression of DNA replication initiation is required to prematurely activate mitotic kinases and to advance mitotic entry. For instance, CDC7 inhibition or CDC6 degradation alone postpones mitotic entry, while combined treatment advances mitotic entry (Figure 3). This suggests that a threshold of replication activity exists, under which mitotic entry is not prevented. DNA replication in human cells is performed by thousands of replication forks (Chagin et al., 2016). How many replication forks are required to reach this threshold is not clear. However, it is likely that a single replication fork is not sufficient to block mitotic entry as Plk1 activation is observed in the presence of occasional weak PCNA-cb foci (Akopyan et al., 2014) and DNA synthesis at fragile sites has been detected in mitotic cells (Minocherhomji et al., 2015, Pedersen et al., 2015).

While complete DNA replication is critical for proper chromosome segregation in mitosis, DNA replication per se is not a prerequisite for mitotic entry. Yeast mutants lacking CDC6, CDT1, ORC2, RFC5, CDC45, or CDC7/DBF4 enter mitosis with unreplicated chromatids (Dowell et al., 1994, Hofmann and Beach, 1994, Liang et al., 1995, Piatti et al., 1995, Sugimoto et al., 1996). Whether mitotic entry in these studies depends on gene-specific functions independent of DNA replication initiation has been debated (Bueno and Russell, 1992). Given that all these proteins have in common to affect initiation of DNA replication, we consider that the most likely explanation involves initiation of DNA replication. In support of this notion, we detect premature mitotic kinase activation and shorter cell cycles in the absence of DNA replication origin licensing and/or origin firing in human cells.

Once initiated, CDK2 activity will promote both DNA replication and stimulate CDK1 and PLK1 activity (Fu et al., 2008, Gheghiani et al., 2017). However, at the same time, CDK1 and PLK1 are restricted by DNA replication, constituting an incoherent feedforward loop (Figure 7). Given that CDK1 activation causes DNA damage during DNA replication, there is a possibility that the feedforward loop is complemented by feedback between CDK1 and DNA replication, ensuring a continuous low level of checkpoint activation. We note that a feedforward system not only ensures that mitosis is avoided until DNA replication is completed but also can ensure that mitotic kinase activation commences immediately after S phase, ensuring a tight coupling between S phase and mitosis.

The feedforward loop between CDK2, DNA replication, and mitotic kinase activation contributes to genome stability in two ways. First, it ensures that mitosis is delayed until the bulk of DNA is duplicated. Second, it prevents DNA replication stress and CDK1-mediated DNA damage. In this sense, complete deregulation of the feedforward loop likely is lethal, whereas slight perturbations have the possibility to contribute to CDK-dependent DNA lesions in S phase (Lecona and Fernández-Capetillo, 2014, Szakal and Branzei, 2013, Zhang and Hunter, 2014) and might promote the occurrence of underreplicated DNA in mitosis (Lukas et al., 2011, Minocherhomji et al., 2015). Such perturbations may not only have consequences for mitosis but also for G1 duration and cell fate of daughter cells (Arora et al., 2017, Barr et al., 2017).

## STAR★Methods

### Key Resources Table

**Table:** 

REAGENT or RESOURCE	SOURCE	IDENTIFIER
**Antibodies**		

CyclinA2	Santa Cruz	sc-751
CyclinA2	Cell Signaling	4656
PLK1	Abcam	ab14210
pTCTP	Cell Signaling Technology	5251
pH2AX	Cell Signaling Technology	2577
pCyclinB1	Abcam	ab55184
pLaminA/C	Cell Signaling Technology	2026
Alexa Fluor 488 Goat anti-Rabbit	Life Technologies	A11008
Alexa Fluor 555 Goat anti-Mouse	Life Technologies	A21422
CDC6	Santa Cruz	sc-9964
CDT1	Santa Cruz	sc-28262
GAPDH	Sigma	G8795
Anti-Rabbit HRP	Abcam	ab6721
Anti-Mouse HRP	Abcam	ab97023

**Chemicals, Peptides, and Recombinant Proteins**		

CHIR-124	Selleck Chemicals	S2683
UCN-01	Sigma	U6508
RO-3306	Calbiochem	217699
NU6140	Calbiochem	238804
Roscovitine	Selleck Chemicals	S1153
CDK1/2 inhibitor III	Millipore	217714
SB202190	Selleck Chemicals	S1077
BI2536	Selleck Chemicals	S1109
GSK461364	Selleck Chemicals	S2193
XL-413	Selleck Chemicals	S7547
BMS-650032	Adooq Bioscience	A11295
3-Indoleacetic acid	Sigma-Aldrich	I3750
Doxycycline	Sigma-Aldrich	D3447

**Experimental Models: Cell Lines**		

BJ	ATCC	CRL-2522
U2OS	Rene Medema (NL)	N/A
Human hTERT-RPE1	Rene Medema (NL)	N/A
Human hTERT-RPE1 p53KO	Libor Macurek (CZ)	N/A

**Oligonucleotides**		

CDC6 siRNA SMARTpool ON-TARGET plus	Dharmacon	003233
Non-targeting siRNA SMARTpool ON-TARGET plus	Dharmacon	001810
CDC45 siRNA SMARTpool ON-TARGET plus	Dharmacon	003232
CDT1_7 siRNA Flexitube	QIAGEN	SI04142250
CDT1_8 siRNA Flexitube	QIAGEN	SI04159477

**Recombinant DNA**		

pCCC-TagRFP (PCNAcb)	Chromotek	N/A
pCSII-EF-Cdk2probe	Sabrina Spencer (US)	N/A
pRetroX-SG2M-Red	Clontech	631465
hCDC6_mAID_SMASh_T2A_Neo	This paper	N/A
pROSA26-DV1_OsTIR	This paper	N/A
PX458_Cas9_GFP	Addgene	48138
PX458_Cas9_GFP_hCDC6	This paper	N/A

**Software and Algorithms**		

ImageJ 1.51h	ImageJ	https://imagej.nih.gov
MATLAB R2015b	MathWorks	https://www.mathworks.com
CellProfiler 2.2.0	CellProfiler	http://cellprofiler.org

### Contact for Reagent and Resource Sharing

Further information and requests for resources and reagents should be directed to and will be fulfilled by the Lead Contact, Arne Lindqvist (arne.lindqvist@ki.se).

### Experimental Model and Subject Details

Source of cell lines used in this study is reported in the Key Resources Table.

### Method Details

#### Cell Culture

Human hTERT-RPE1 (hereafter referred to as RPE), U2OS and BJ cells were cultured in an ambient-controlled incubator at 37°C and 5% CO2. Early passage BJ cells were purchased from ATCC (CRL-2522) and examined prior to population doubling 10. RPE and U2OS cells were a kind gift from Dr. René Medema. All cell lines were regularly tested for mycoplasma. RPE cells were cultured using DMEM-F12 GlutaMAX (Invitrogen) supplemented with 1% Pen/Strep (HyClone) and 10% heat-inactivated FBS (HyClone). U2OS and BJ cells were cultured using DMEM GlutaMAX (Invitrogen) supplemented with 1% Pen/Strep (HyClone) and 6% or 10% heat-inactivated fetal bovine serum (FBS, HyClone), resp.

#### Plasmids and Cell lines

Reporter plasmids pCCC-TagRFP (Chromotek, PCNA-cb), pCSII-EF-Cdk2probe (Spencer et al., 2013) or pRetroX-SG2M-Red (Clontech, 631465) were used to obtain stable cell lines by random plasmid integration. Plasmid transfection was performed using LipofectAMINE 2000 Reagent (Life Technologies) or standard calcium phosphate precipitation. Stable polyclonal populations were selected by flow cytometry using BD-FACSAria II and correct expression patterns were validated by live-cell microscopy.

TP53 knockout RPE cells were from Silva Cascales et al. (2017). Stable *Os*TIR expression was established by CRISPR-mediated integration: Transient GFP+ cells were sorted 3 days post transfection of pROSA26-DV1_OsTIR and PX458_Cas9_GFP (Addgene 48138) and subsequently subjected to bleomycin selection. For inducible expression of OSTir1 we used a previously described construct (Natsume et al., 2016) that was combined with a bleomycin selection cassette and cloned into a Rosa26 targeting construct to generate pROSA26-DV1_OsTIR. To target the human Rosa26 locus we used the following gRNA sequence: 5′ GACCTGCTACAGGCACTCGT 3′ cloned into pPV576_gRNA_ROSA26. Subsequent double degron tagging at the human CDC6 locus was established through CRISPR-mediated targeting: First a CDC6 guide sequence was cloned into PX458_Cas9_GFP (Addgene 48138) using BbSI sites and two annealed oligos CACCGC CAGCTG AATACT TTCGGG and AAACCC CGAAAG TATTCA GCTGGC. Second, a tagging vector was generated named hCDC6_mAID_SMASh_T2A_Neo, by cloning CDC6 homology arms (gBlock IDT) into a mAID_SMASh_T2A_Neo backbone using SbfI and NotI sites. The mAID_SMASh_T2A_Neo backbone was constructed via Gibson assembly cloning.

The resultant PX458_Cas9_GFP_hCDC6 and hCDC6_mAID_SMASh_T2A_Neo vectors were sequence-verified and transfected into *Os*TIR-RPE^P53KO^ cells. Three days post transfection GFP+ cells were sorted and subjected to Geneticin selection (G418; GIBCO). Clonal cell lines were established by limiting dilution in a 96-well plate. Successful Cdc6 degron-tagging in single clones was verified by western blot and degron-induced growth arrest.

#### RNA Interference and Inhibitors

Knockdown studies were performed using SMARTpool scrambled control siRNA (Dharmacon), SMARTpool ON-TARGET plus CDC6 siRNA (Dharmacon), SMARTpool ON-TARGET plus CDC45 siRNA (Dharmacon), FlexiTube Hs_CDT1_7 (QIAGEN) and FlexiTube Hs_CDT1_8 (QIAGEN). siRNAs were transfected at 20nM concentration using HiPerFect reagent (QIAGEN) and OptiMEM (Invitrogen). The following small-molecule inhibitors were used at the indicated final concentrations: 50 nM CHIR-124 (CHK1 inhibitor; Selleck Chemicals), 30nM UCN-01 (CHK1 inhibitor; Sigma), 5 uM RO-3306 (Cdk1 inhibitor; Calbiochem), 10 uM NU6140 (Cdk2 inhibitor; Calbiochem), 25uM Roscovitine (Cdk1/2 inhibitor; Selleck Chemicals), 3uM CDK1/2 inhibitor III (Millipore),10 uM SB202190 (p38 inhibitor; Selleck Chemicals), 50 nM BI2536 (PLK1 inhibitor, Selleck Chemicals), 200nM GSK461364 (PLK1 inhibitor; Selleck Chemicals), 2.5 mM Thymidine (Sigma Aldrich), 15 uM XL-413 (CDC7 inhibitor; Selleck Chemicals), 100 nM BMS-650032 (Asunaprevir; Adooq Bioscience), 50 uM 3-Indoleacetic acid (Auxin; Sigma Aldrich), 1ug/ml Doxycycline (Sigma Aldrich).

#### Live-Cell Microscopy and Quantitative Immunofluorescence

For time-lapse microscopy of RPE CDC6^d^ cells (Figure 2D), transmitted light images were acquired every 10 min using a Leica DMI6000 Imaging System with 20x (NA 0.4) air objective at 37°C and 5% CO2. Images were analyzed using ImageJ and cells were tracked manually. For live cell fluorescence imaging experiments, 6000-9000 cells were seeded in 96-well imaging plates (BD Falcon) in DMEM with 1% Pen/Strep and 6%–10% FBS and at least 6 hr prior to imaging the medium was changed to Leibowitz-15 (Invitrogen) with 1% Pen/Strep and 6%–10% FBS. Images were acquired at 37°C at 20-45min intervals using ImageXpress microscope (Molecular Devices) 20X objective (NA 0.45) and analyzed using custom ImageJ or MATLAB scripts (Akopyan et al., 2014, Hukasova et al., 2012). PLK1 FRET was measured using ImageJ (Akopyan et al., 2014, Hukasova et al., 2012), using rolling ball background subtraction, 3x YFP signal multiplication and RatioPlus plugin (background 0 and clipping values 25-50). Normalized PCNA-cb intensity variation (i.e., standard deviation of nuclear RFP signal divided by median nuclear RFP intensity) is used as a surrogate measurement for DNA replication; the last few bright PCNA foci at the end of S-phase cause a characteristic peak in PCNA-cb intensity variation (Figures 1C and 4C). CDK1/2 activity probe was analyzed using ImageJ; nuclear ROIs were selected manually and ratio between nuclear YFP intensities and cytoplasmic YFP intensities was determined using custom ImageJ macro’s and Excel (see Data S1 for example macro). For quantitative IF experiments 6000-9000 cells were seeded in 96-well imaging plates (BD Falcon) in DMEM with 1% Pen/Strep and 6%–10% FBS and after treatment washed in DPBS (GIBCO), fixed in formalin (Sigma Aldrich) for 7 min, permeabilised in cold methanol (Sigma Aldrich) for 2 min and finally incubated in blocking media (TBS supplemented with 0.1% Tween20 and 2% bovine albumin serum) for 1 hr at room temperature. For pulse-chase experiments or DNA replication measurements 10mM EdU (5-ethynyl-2′-deoxyuridine, Molecular probes) was added to live cells prior to fixation. Fixed samples were incubated with primary antibodies in blocking media overnight at 4°C, washed in DPBS (GIBCO) and incubated with secondary antibodies and DAPI for 1h at room temperature. Samples were stored in PBS and in case of DNA replication measurements EdU-Click chemistry was performed by incubation in 100mM Tris, 1mM CuSO4, 100mM ascorbic acid and fluorescent dye azide (#A10277, Invitrogen) for 1h at room temperature. Images were acquired at room temperature using ImageXpress microscope (Molecular Devices) 20X objective (NA 0.45) and analyzed using custom Cell profiler pipelines. To assess kinetics and cell cycle stages from quantitative IF data, cells were ordered and distributed linearly based on increasing nuclear Cyclin A2 intensities and normalized DAPI levels (Akopyan et al., 2016). For RPE and BJ cells in Figure 1C, cells containing low DAPI levels (corresponding to under mid S-phase levels) were sorted based on the ratio between nuclear size and roundness, and remaining cells were sorted by Cyclin A2 content.

#### Antibodies

The following antibodies were used for IF and diluted in TBS supplemented with 0.1% Tween20 and 2% BSA: CyclinA2 (1:400; #sc-751; Santa Cruz), CyclinA2 (1:400; #4656; Cell Signaling), PLK1 (1:400; ab14210; Abcam), pTCTP (1:400; #5251; Cell Signaling), pH2AX (1:800; #2577; Cell Signaling), pCyclinB1 (1:400; #ab55184; Abcam), pLaminA/C (1:400; #2026; Cell Signaling), Alexa Fluor 488-Goat anti-Rabbit (1:2000; #A11008 Life Technologies) and Alexa Fluor 555-Goat anti-Mouse (1: 2000; #A21422 Life Technologies). The following antibodies were used for WB and diluted in TBS supplemented with 0.1% Tween20 and 5% milk powder: CDC6 (1:400; #sc-9964 Santa Cruz), CDT1 (1:400; #sc-28262 Santa Cruz), GAPDH (1:20000; #G8795; Sigma), anti-Rabbit HRP (1:2000; #ab6721; Abcam) and anti-Mouse HRP (1:2000; #ab97023; Abcam).

### Quantification and Statistical Analysis

Statistical analysis was performed using the Student’s t test. Quantitative IF studies included >200 cells per conditions and represent at least two independent experiments. Qualitative example images are representative for >10 independent biological replicates. Information about the statistical tests and the number of cells analyzed is provided in the figure legends. For all experiments, samples were not randomized and the investigators were not blinded to the group allocation during experiments and outcome assessment.

### Data and Software Availibilty

Original imaging data have been deposited to Mendeley Data and are available at https://doi.org/10.17632/47s44zbm7c.1.

## Supplemental Information

Document S1. Figures S1–S7

Data S1. ImageJ Macro Used for Measurement of CDK Activity Reporter, Related to STAR Methods

Document S2. Article plus Supplemental Information
