# Supplementary material for: DNA Replication Determines Timing of Mitosis by Restricting CDK1 and PLK1 Activation
Source: Mol Cell. 2018 Jul 5;71(1):117–128.e3. doi: 10.1016/j.molcel.2018.05.026 (PMC6039720; doi:10.1016/j.molcel.2018.05.026)
Supplement: Document S1. Figures S1–S7 [file mmc1.pdf]

Molecular Cell, Volume *71*

Supplemental Information

DNA Replication Determines Timing of Mitosis by Restricting CDK1 and PLK1 Activation

Bennie Lemmens, Nadia Hegarat, Karen Akopyan, Joan Sala-Gaston, Jiri Bartek, Helfrid Hochegger, and Arne Lindqvist

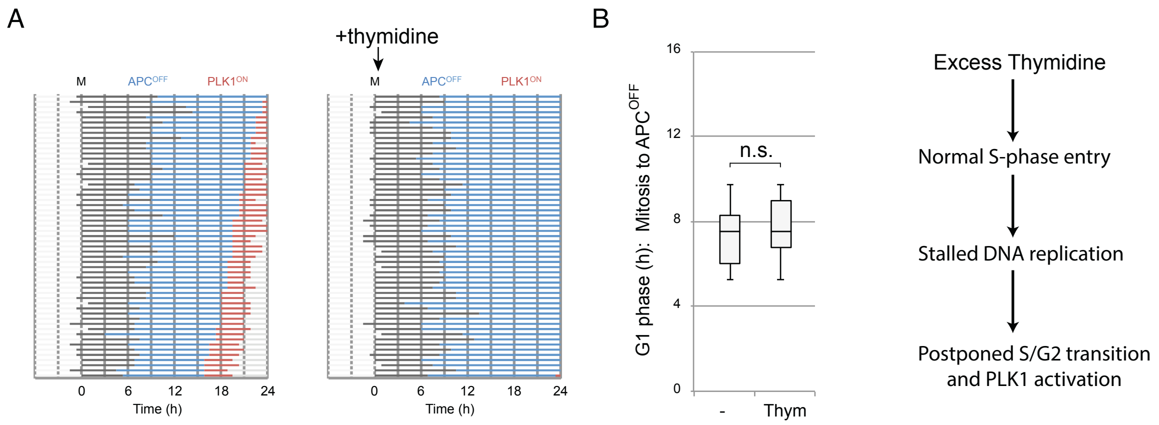

Figure S1. Related to Figure 1; Stalled DNA replication postpones PLK1 activation
(A) Asynchronous U2OS cells carrying PLK1-FRET and APC/CCdh1 substrate reporters were mock treated or treated with excess thymidine (2.5mM). For each condition 50 single cells we selected that were at mitosis upon treatment (+/- 1 h) and appearance of the APC/CCdh1 substrate (APCOFF) or nuclear PLK1 activity (PLK1ON) was scored during the following 24 hours. (B) Boxplot depicts G1 phase duration of 50 single cells as determined by the time observed between mitosis and appearance of the APC/CCdh1 substrate (APCOFF). Box plots indicate 10, 25, 50, 75, and 90th percentile. n.s. indicates $p<0.5$, students t-test.

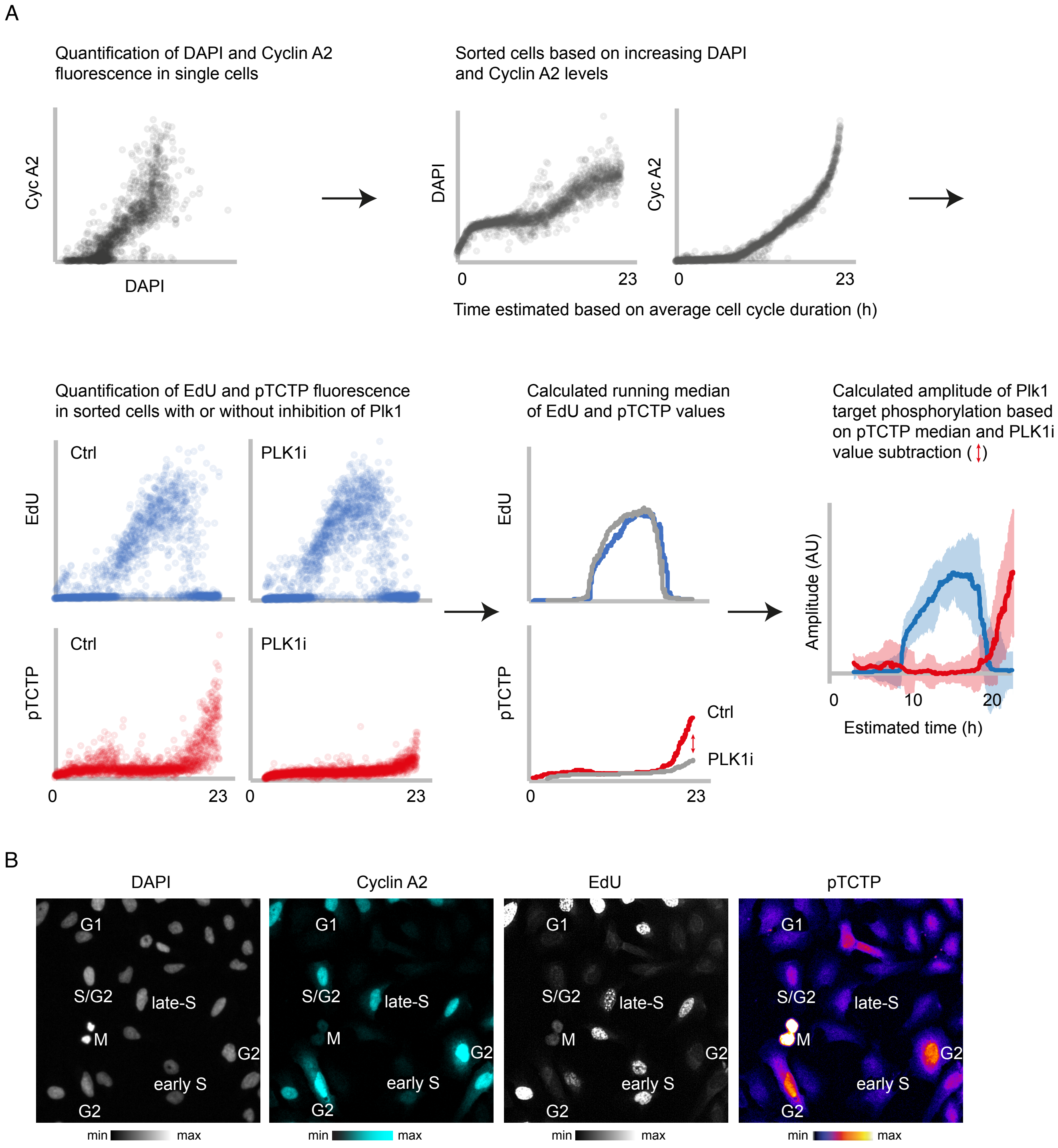

Figure S2. Related to Figure 1; Workflow assessing DNA replication and phosphorylation kinetics from fixed cells (A) Stepwise description of the workflow used to asses PLK1 target phosphorylation throughout the cell cycle based on high-content imaging of fixed cells. Validation and broad use of this approach is described in Akopyan et al. 2014. Cells were fixed after a 1h EdU pulse and analyzed as indicated; graphs depict U2OS data. (B) Example images of U2OS cells described in (A). Representative cells for each cell cycle phase are indicated. Please note that PLK1 target phosphorylation (pTCTP) is enhanced in G2 cells (Edu negative, nuclear and cytoplamic CyclinA2 positive cells) and peaks when cells enter mitosis, which mirrors PLK1 FRET probe phosphorylation (Akopyan et al. 2014).

Lemmens_Figure S3

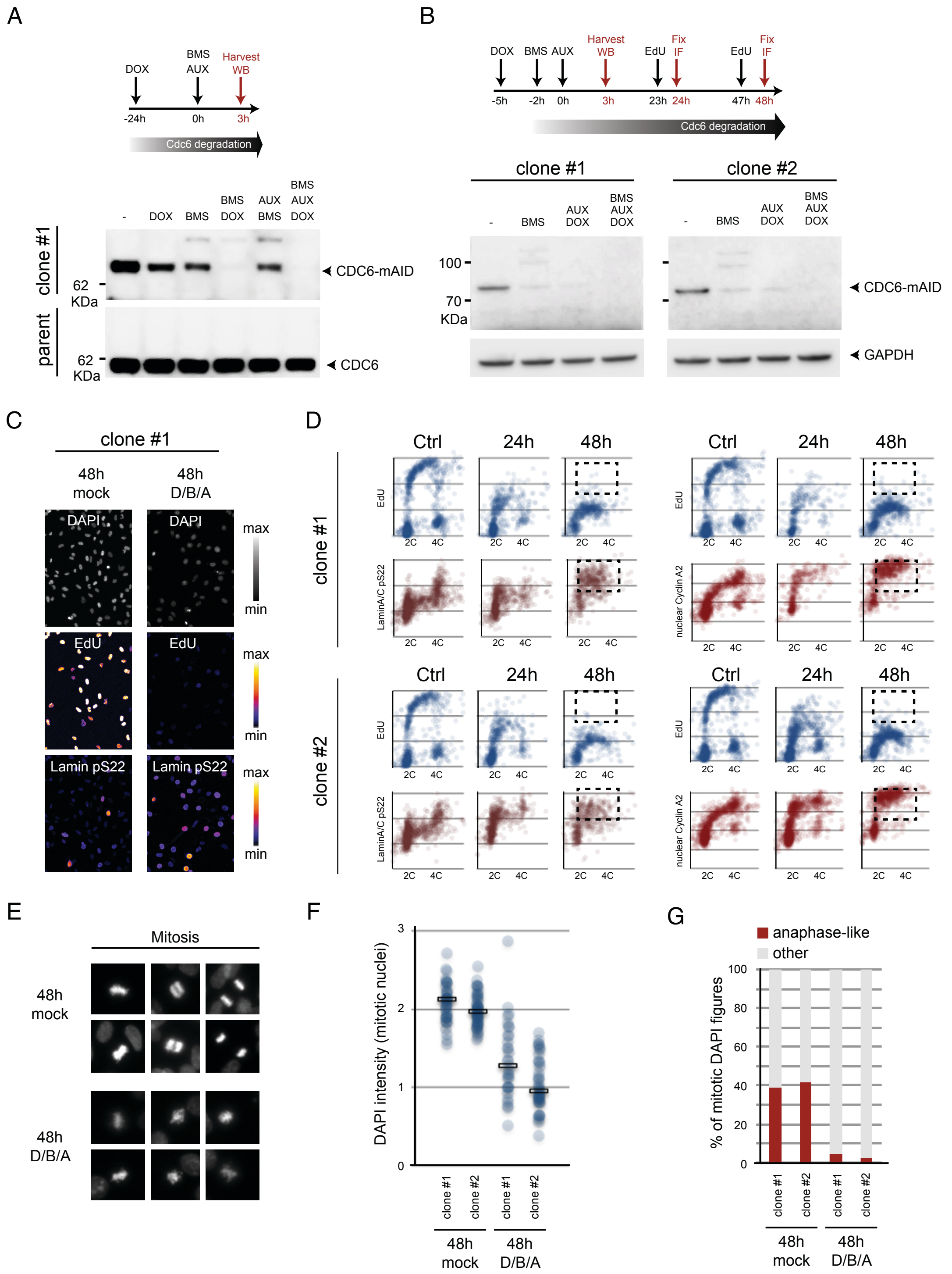

Figure S3. Related to Figure 2; CDC6 degradation in RPE CDC6d cells prevents DNA replication but allows cell cycle progression (A). Western blot analysis of CDC6 degradation after treatment as indicated in upper panel. RPE CDC6d cells and parental RPE cells were pre-treated with 1ug/ml doxycycline (DOX) for 24h to induce OsTIR expression and exposed to 1uM asunaprevir (BMS) and 0.5 mM auxin (AUX) for 3h. Cells were harvested and cell lysates were probed for CDC6 protein. We noted that OsTIR expression can cause AUX-independent CDC6-mAID degradation and thus minimized DOX pretreatment in all other setups described in this study. (B) Two independent clonal RPE CDC6d cell lines were treated with 1ug/ml DOX, 100nM BMS and 50uM AUX and harvested as indicated in upper panel. Cell lysates were probed for CDC6 protein and GAPDH for loading. Please note enhanced CDC6 degradation by combining two degron systems. (C) RPE CDC6d cells were mock-treated or treated with DOX, BMS and AUX as depicted in (B) and fixed for immunofluorescence analysis 48h post AUX addition. DAPI, EDU and Lamin A/C pS22 intensities are false-colored as indicted on the right. Please note anti-correlation between EdU incorporation and CDK-target phosphorylation. (D) Two independent clonal RPE CDC6d lines were mock-treated (Ctrl) or treated with DOX, BMS and AUX as depicted in (B) and fixed 24h or 48h post AUX addition. Graphs depict DAPI intensities against nuclear EdU, Lamin A/C pS22 or CyclinA2 intensities of >600 cells per condition. Dashed box highlights population of S/G2 cells having high levels of Lamin A/C pS22 or CyclinA2 yet lacking EdU incorporation (E) Examples of mitotic DAPI figures of RPE CDC6d cells treated as in (C). (F) Quantification of DAPI intensities of mitotic cells after treatment as in (C). (G) Quantification of anaphase DAPI figures among mitotic cells after treatment as in (C).

Lemmens_Figure S4

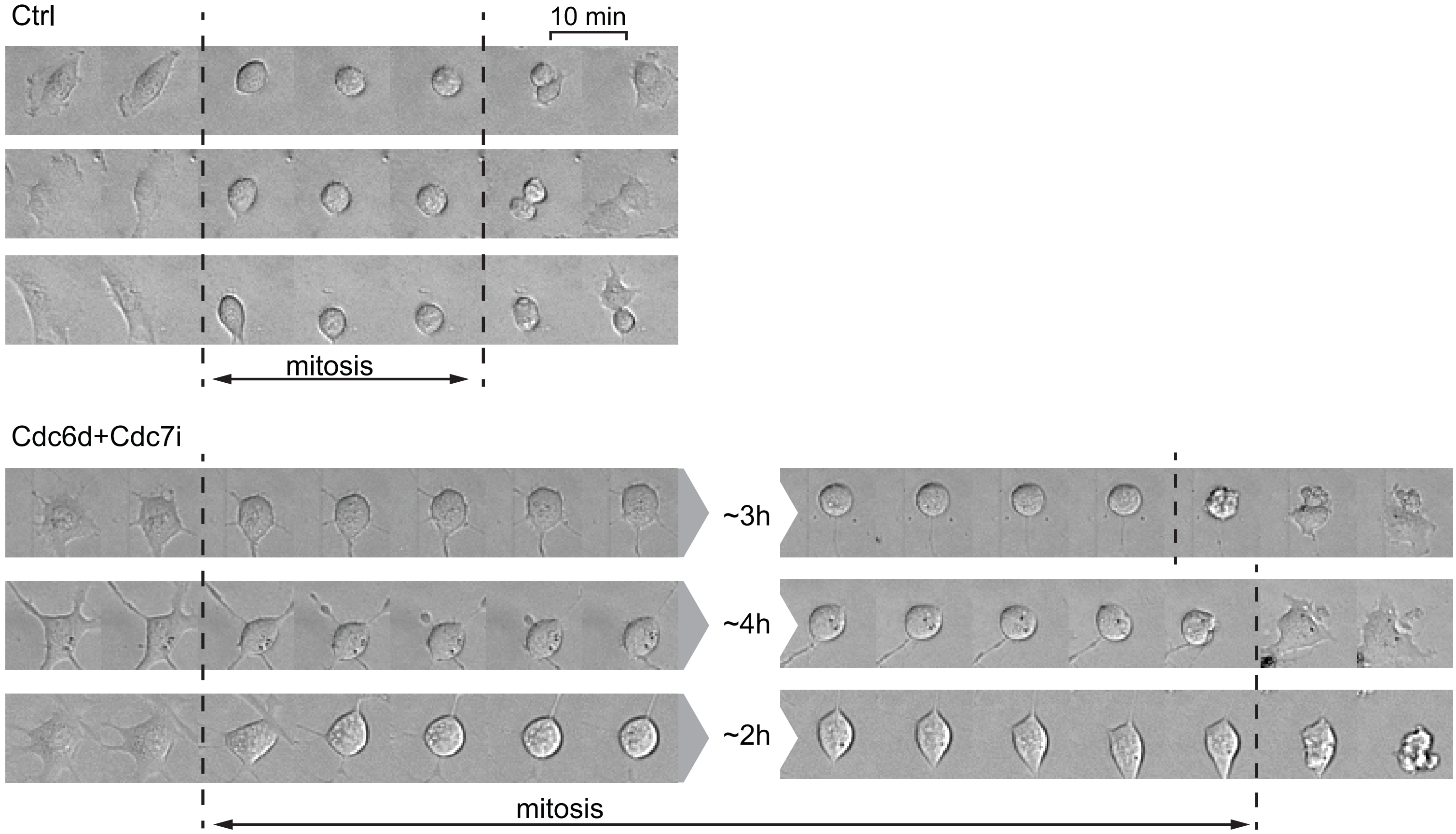

Figure S4. Related to Figure 3; Examples of mitosis in three control and three DOX/BMS/AUX/Cdc7i treated cells, as quantified in Fig 3D. Time between images 10 min. Mitotic entry was estimated primarily by cell rounding, and when possible confirmed by disappearance of nucleoli and nuclear border (dotted line to left). The duration of mitosis was estimated until anaphase onset, the cell settling down, or dying (dotted line to right). A movie including both the first mitosis (shortly after drug addition) and the second mitosis visualised here is available as Movie S1.

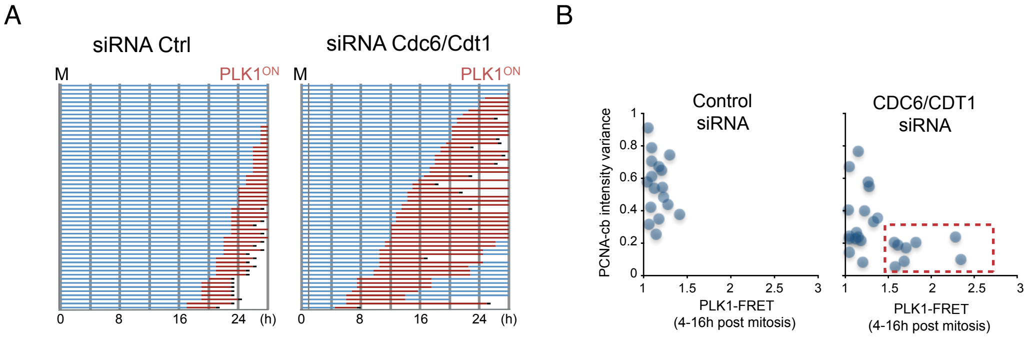

Figure S5. Related to Figure 4; Impaired DNA replication licensing causes premature PLK1 activation (A) Asynchronous U2OS cells carrying PLK1-FRET and APC/CCdh1 substrate reporters were mock treated or treated with excess thymidine (2.5mM). For each condition 50 single cells we selected that were at mitosis upon treatment (+/- 1 h) and appearance of the APC/CCdh1 substrate (APCOFF) or nuclear PLK1 activity (PLK1ON) was scored during the following 24 hours. (B) Boxplot depicts G1 phase duration of 50 single cells as determined by the time observed between mitosis and appearance of the APC/CCdh1 substrate (APCOFF). Box plots indicate10, 25, 50, 75, and 90th percentile. n.s. indicates $p<0.5$, students t-test.

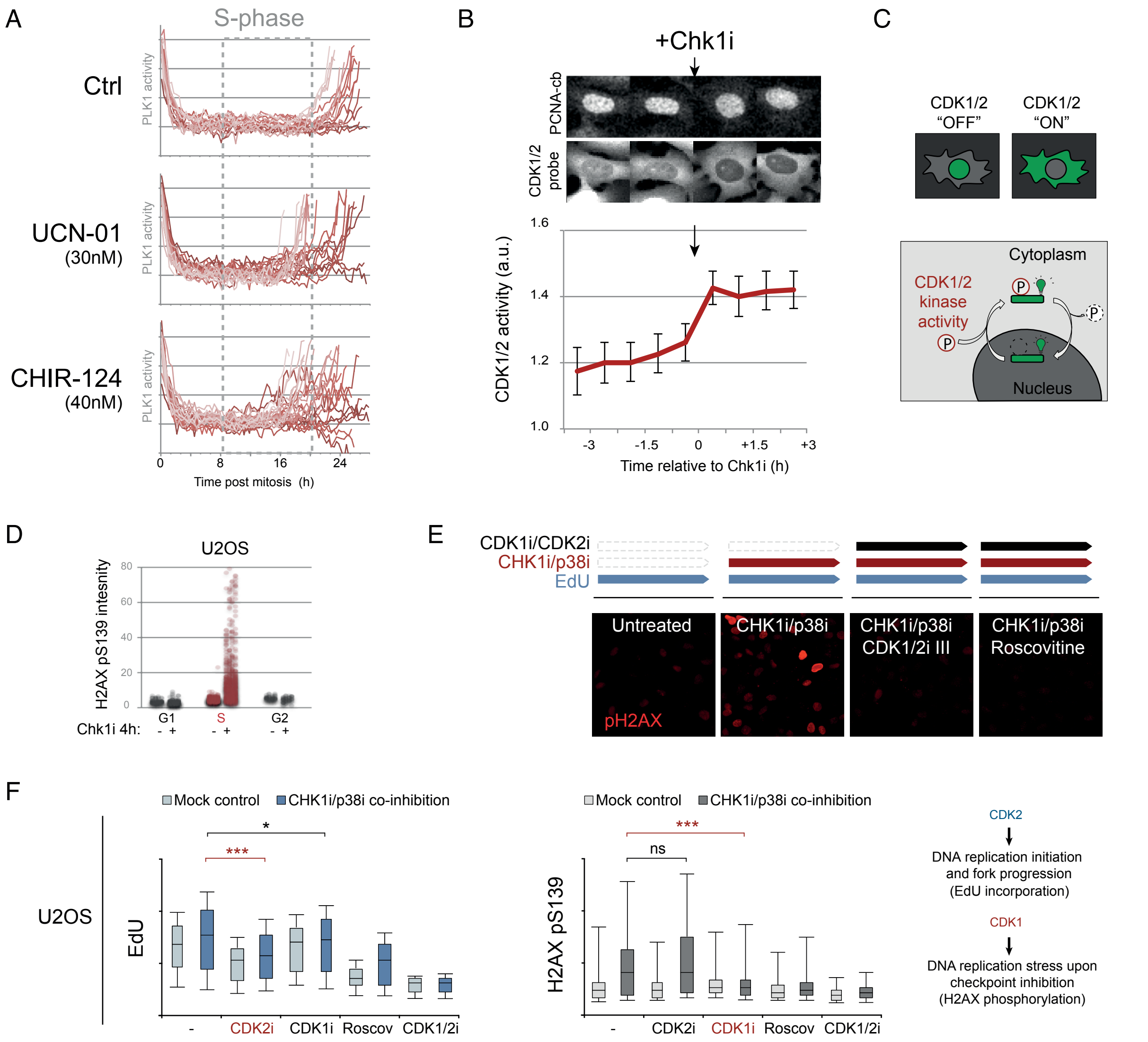

Figure S6. Related to Figure 5; CHK1 and p38 limit PLK1 activation and CDK1-dependent replication stress (A). U2OS cells expressing PLK1-FRET were followed by time-lapse microscopy. Cells that were +/- 1h from mitosis upon addition of indicated inhibitors were selected for analysis. Panels show quantification of PLK1-FRET of 20-25 single cells per condition. The approximate position of S-phase based on time after mitosis is indicated. (B). U2OS cells expressing CDK1/2 sensor and containing visible PCNA-cb foci were followed every 45 min by time-lapse microscopy. Graph shows average and SD of CDK1/2 activity of 25 cells, relative to time point of addition of CHIR-124. (C). Schematic of CDK1/2 sensor. Top, nuclear/cytoplasmic ratios of probe intensity reflect CDK1/2 activity. Bottom, CDK1/2 driven phosphorylation causes cytoplasmic translocation of the probe (Spencer et al., 2013) (D). U2OS cells were treated with CHIR-124 (CHK1i) as indicated. To define S-phase, cells were incubated with EdU 1h prior to fixation. Panels show quantifications of high-content microscopy for H2AX pS139 in single cells. Cell populations were separated according to DAPI and EdU intensity. (E). RPE cells were treated with EdU, CHIR-124 (CHK1i), SB202190 (p38i), CDK1/2 inhibitor III, and/or Roscovitine as indicated for 4h. Images show staining for H2AX pS139. (F). U2OS cells were treated with CHIR-124 (CHK1i), NU6140 (CDK2i), RO3306 (CDK1i), CDK1/2 inhibitor III (CDK1/2i), and Roscovitine as indicated for 4h. Before fixation, cells were incubated for 1h with EdU. Box plots show 90, 75, 50, 25, and 10th percentiles of EdU signal or H2AX pS139 signal of EdU positive cells, as assessed by high-content microscopy. * indicates p=0,04, *** indicates p<0.001 and ns indicates p>0.1, student's t-test.

Lemmens_Figure S7

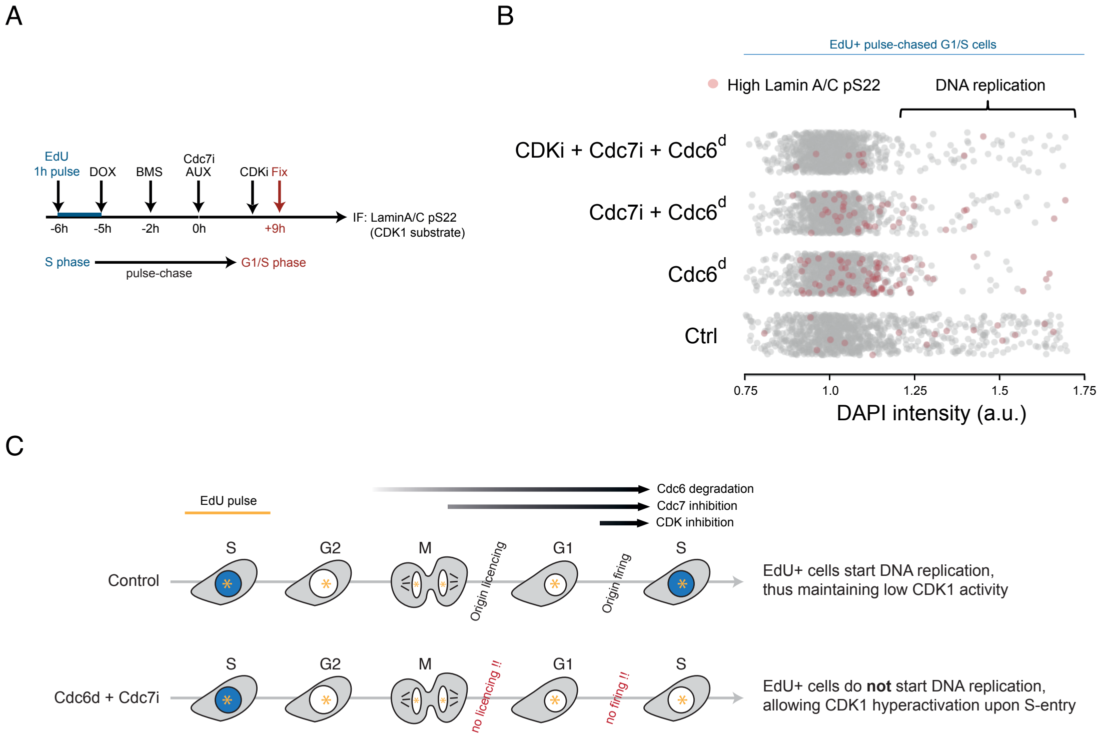

Figure S7. Related to Figure 6; Preventing DNA replication causes CDK hyperactivation at G1/S transition (A) Schematic of experimental setup. EdU pulse-chase is used to mark cells that are expected to be devoid of CDC6 in the next G1 phase; the time CDC6 is required for origin licensing (B) Dot plot depicts single RPE CDC6d cells treated as outlined in (A) and analyzed by high-content microscopy. Only EdU positive cells are analyzed to enrich for cells that were in G2/M phase during induced depletion of CDC6d or addition of CDC7 inhibitor. Graph includes EdU positive cells with low (grey dots) or enhanced (red dots) phosphorylation of Lamin A/C S22, plotted versus DNA content as measured by DAPI intensity (n=1800 cells per condition). Please note that 14 hours post EdU pulse a subset of Ctrl cells has entered S-phase and started to gain DNA content, while maintaining low pLamin A/C pS22 levels. Notably, upon CDC6d depletion (and CDC7 inhibition) a subset of cells fail to gain DNA content and instead show enhanced phosphorylation of Lamin A/C S22. The enhanced phosphorylation of Lamin A/C S22 depends on CDK1/2 activity as it is reversed by addition of CDK1/2 III 30min prior to fixation. (C) Schematic of cell fate of EdU pulse-chased single cells upon Cdc7 inhibition/Cdc6 degradation.
